# Mapping mental healthcare professionals’ journey towards digital mental health adoption: A qualitative study

**DOI:** 10.1192/j.eurpsy.2021.933

**Published:** 2021-08-13

**Authors:** C. Mendes-Santos, F. Nunes, E. Weiderpass, R. Santana, G. Andersson

**Affiliations:** 1 Department Of Culture And Society (ikos), Linköping University, Linköping, Sweden, Portugal; 2 Human-centered Design, Fraunhofer Portugal AICOS, Porto, Portugal; 3 Direction, International Agency for Research on Cancer, Lyon, France; 4 Public Health Research Centre, NOVA National School of Public Health, Lisbon, Portugal; 5 Department Of Behavioural Sciences And Learning, Linköping University, Linköping, Sweden

**Keywords:** Digital Mental Health, Internet interventions, Technology acceptance, Portugal, EU

## Abstract

**Introduction:**

Digital Mental Health holds strategic potential in fulfilling populations’ mental healthcare unmet needs, enabling convenient and equitable access to mental healthcare. However, despite strong evidence of efficacy, uptake by mental healthcare providers remains low and little is known about factors influencing adoption and its interrelationship throughout the Digital Mental Health adoption process.

**Objectives:**

This study aimed at gaining in-depth understanding of factors influencing adoption and mapping its interrelationship along different stages of the Digital Mental Health adoption process.

**Methods:**

This work adopted a qualitative approach consisting of in-depth semi-structured interviews with 13 mental healthcare professionals, including both psychologists and psychiatrists. The interviews were transcribed and analysed thematically, following Braun and Clarke’s method.

**Results:**

In this communication, we will describe how digital technology is currently used by clinicians to deliver mental healthcare. We identify potential factors influencing Digital Mental Health adoption and characterize the different identified stages inherent to this appropriation process: i) Pondering appropriate use; ii) Contractualizing the therapeutic relationship; iii) Performing online psychological assessment; iv) Adapting and/or developing interventions; v) Delivering Digital Mental Health interventions; and vi) Identifying training unmet needs. A discussion on how different factors and its interrelationship impact the adoption process will also be performed.

**Conclusions:**

By characterizing mental healthcare providers journey throughout the Digital Mental Health adoption process, we intend to inform ecosystem stakeholders, such as researchers, policy makers, societies and industry, on key factors influencing adoption, so policies, programs and interventions are developed in compliance with this knowledge and technology is more easily integrated in clinical practice.

